# Point-of-care ultrasonography for diagnosis of purulent pericarditis postappendectomy: a case report

**DOI:** 10.31744/einstein_journal/2024RC0962

**Published:** 2024-11-19

**Authors:** José Alexandre, Emanuel Pinheiro Esposito, Marcus Gomes Bastos, Cenilde da Costa Araújo, Luan Moraes Ferreira, Apollo Vinícius Fernandes Neves

**Affiliations:** 1 Universidade do Estado do Pará Faculdade de Medicina Santarém PA Brazil Faculdade de Medicina, Universidade do Estado do Pará, Santarém, PA, Brazil.; 2 Universidade Federal de Juiz de Fora Faculdade de Medicina Juiz de Fora MG Brazil Faculdade de Medicina, Universidade Federal de Juiz de Fora, Juiz de Fora, MG, Brazil.

**Keywords:** Pericarditis, Pericardial effusion, Appendectomy, Suppuration, Critical care, Ultrasonography

## Abstract

Purulent pericarditis is rare condition in the modern era of antibiotics. However, it is a serious condition as it has an accelerated progression and is difficult to diagnose due to its nonspecific clinical presentation, resulting in high mortality. Herein, we present a case in which a 36-year-old male patient with otherwise unremarkable medical history developed abdominal sepsis complicated by purulent pericarditis post-appendectomy. While the initial clinical presentation was not compatible with the classic signs of purulent pericarditis, the diagnosis was made using electrocardiography (ST elevation/PR depression) and point-of-care ultrasonography (pericardial effusion). The condition was successfully managed with pericardial drainage and broad-spectrum antibiotics. The present case reinforces and reiterates the need for high diagnostic suspicion and careful clinical reasoning in the diagnosis of purulent pericarditis. Furthermore, it highlights the applicability of point-of-care ultrasonography in the diagnosis of the same.

## INTRODUCTION

Purulent pericarditis, inflammation of the pericardial layers caused by localized bacterial infection, is a rare condition in the modern antibiotic era, accounts for less than 1% of all pericarditis cases,^(1)^ with an incidence of 1 in 18,000 hospitalized patients.^(2)^ However, it has a high mortality rate, reaching 100% among untreated patients. Moreover, 40% of patients who receive adequate treatment die of associated complications, such as constrictive pericarditis, cardiac tamponade, or septic shock.^(1,3)^ The main risk factors for the development of purulent pericarditis are immunosuppression, alcohol abuse, and chest trauma.^(3)^

Herein, we present the case of a male patient who developed purulent pericarditis post-appendectomy and was appropriately diagnosed and successfully treated in an intensive care unit (ICU).

## CASE REPORT

A 36-year-old male was admitted to our ICU on seven days post-appendectomy after being transferred from the emergency department of a hospital in another city with a diagnosis of abdominal sepsis. On arrival, the patient was on mechanical ventilation and deep sedation with fentanyl and midazolam. Vital sign assessment revealed an axillary temperature of 38°C, heart rate of 98 bpm, and blood pressure of 75/53 mmHg. Lung auscultation revealed decreased vesicular breath sounds at the right base, and cardiac auscultation showed no abnormalities. The surgical wound was clean and showed no signs of inflammation. Abdominal examination revealed no changes. Laboratory tests indicated the following: hemoglobin, 12.3g/dL; leukocytes, 13,000/mm³; platelets, 400.00mm³; total bilirubin, 2.4mg/dL; direct bilirubin, 1.7mg/dL; creatinine, 1.2 mg/dL; urea, 163 mg/dL.

**Figure 1:**
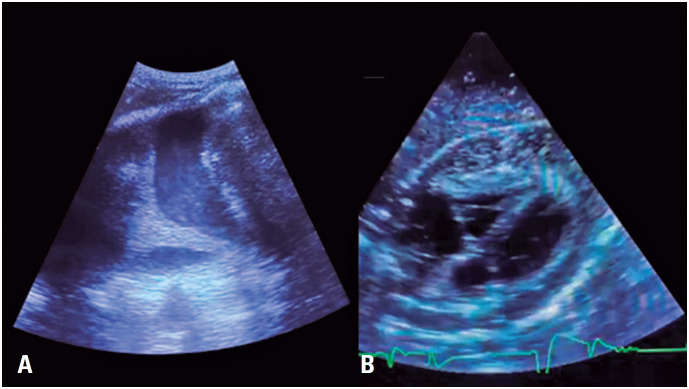
Point-of-care ultrasonography revealed right septated pleural effusion (A) and pericardial effusion with fine internal echoes (B)

Electrocardiography (ECG) revealed ST segment elevation in multiple leads and PR segment depression in the precordial leads. Point-of-care ultrasonography (PoCUS) revealed a significant septated pleural effusion on the right side and a large pericardial effusion with signs of right ventricular restriction and fine internal echoes, suggestive of empyema (Figure 1).

Computed tomography (CT) of the abdomen and pelvis revealed parenchymal edema and a heterogeneous lace-like enhancement pattern in the liver, indicating congestion, as well as approximately 100mL of free viscous fluid in the pelvic region with no organized walls. These findings were consistent with an abdominal infection.

**Figure 2:**
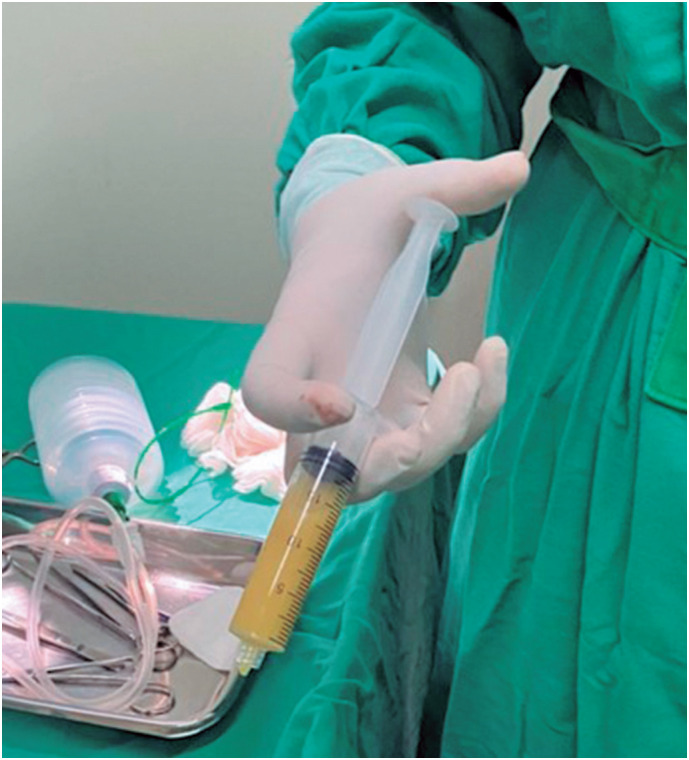
Purulent fluid drained from the pericardial cavity via ultrasound-guided pericardiocentesis

Ultrasound-guided pericardiocentesis was performed, resulting in the drainage of approximately 370mL of thick, purulent, and foul-smelling fluid, consistent with purulent pericarditis (Figure 2). Subsequently, a pericardial drain was placed, yielding approximately 735mL of fluids over a four-day period. Furthermore, a thoracostomy was performed, extracting approximately 1200mL of fluid resembling pericardial effusion over six days. After the procedure, broad-spectrum antibiotic therapy was initiated with vancomycin and piperacillin-tazobactam.

Analysis of the pericardial fluid revealed the following: abundant leukocytes; polymorphonuclear cells, 96% red blood cells, 17.5/mm³; amylase, 15U/L; total proteins, 2.9g/dL; glucose, 40mg/dL; lactate dehydrogenase, 2115U/L; pH 7.0; negative fungal and acid-fast bacillus tests; and bacterioscopy with frequent gram-positive coccobacilli. The pleural fluid had similar biochemical characteristics, including the presence of gram-positive coccobacilli. Further, tests for hepatitis B, hepatitis C, and HIV were negative. Although microbial cultures were negative, antibiotic therapy was maintained because of the patient's clinical presentation, characteristics of the drained fluids, results of bacterial tests, and the unavailability of more sophisticated tests to identify pathogens.

The patient exhibited significant clinical improvement in the subsequent days. Extubation was performed four days after admission, and the patient was discharged to the general ward occurred after 8 days. The patient was discharged 25 days after admission with no recurrence.

This study was approved by the Research Ethics Committee of the *Universidade do Estado do Pará* (CAAE: 74963523.0.0000.5168; #6.578.763).

## DISCUSSION

Pericarditis, inflammation of the pericardial layers, is the most common form of pericardial disease in clinical settings.^(1,4)^ It can stem from infections and non-infectious causes, such as autoimmune diseases, drugs, and neoplasms. In developed countries, pericarditis is predominantly linked to viral infections, whereas in developing countries, it is frequently associated with tuberculosis, with episodes caused by other bacterial infections being notably less common.^(1,4)^

Purulent pericarditis, on the other hand, is primarily caused by gram-positive cocci, accounting for up to 33% of the cases. However, the involvement of gram-negative bacilli, such as *Escherichia coli* and *Pseudomonas aeruginosa* has been noted in older adults and debilitated individuals.^(3,5)^ Cases attributed to infections by anaerobic species, including *Clostridium*, *Fusobacterium*, and *Actinomyces* spp., have also been documented.^(3)^ In general, purulent pericarditis occurs secondary to an infection within the thorax, direct inoculation via trauma, or hematogenous spread from distant foci.^(3)^

As noted in the present case, identification of pathogens in blood cultures can be challenging, as most patients receive antibiotics prior to collection of blood and pericardial fluid. Additionally, the presence of atypical microorganisms and the production of autolysin by commensal bacteria, such as *Streptococcus pneumoniae,* contribute to negative cultures,^(6)^ which was also noted in the present case. While polymerase chain reaction offers interesting possibilities for pathogen identification in cases with negative cultures, it has certain limitations with respect to collection technique, low availability, high cost, and lack of information on antimicrobial susceptibility.^(6)^

While the classic clinical presentation of purulent pericarditis includes chest pain, pulsus paradoxus, pericardial friction rub, and fever, more than 2/3 of patients do not exhibit obvious signs on physical examination.^(7,8)^ Similarly, while common ECG alterations include ST elevation/PR depression, electrical alterations, and low-voltage QRS complexes, less than 50% of patient exhibit these signs on ECG.^(2,9)^

Point-of-care ultrasonography is an invaluable and practical diagnostic tool that can identify pericardial effusion with an accuracy of 97.5%. Further, it can be used to guide pericardiocentesis and to verify the effectiveness of the procedure.^(10)^ A pericardial effusion is visualized as an echo-free space between the heart and parietal layer of the pericardium, and the volume of the collection can be estimated based on the distance between the pericardial layers.^(10)^ Serous fluids usually have an anechoic appearance, whereas hemorrhagic and purulent fluids are echogenic.^(10)^ Hematomas and neoplastic diseases manifest as solid pericardial masses.^(10)^ Furthermore, PoCUS allows the accurate identification of signs of cardiac tamponade, a common complication of purulent pericarditis,^(1,3)^ including inferior vena cava distension and cardiac chamber collapse.^(10)^

## CONCLUSION

In summary, we present the case of a patient who developed purulent pericarditis post-appendectomy. While the initial physical examination provided only few clues for the diagnosis as classical signs of purulent pericarditis were not present, the systematic use of point-of-care ultrasonography aided in the diagnosis and pericardial drainage. The present cases reinforces the need for a high degree of suspicion and thorough clinical reasoning for suspected cases of purulent pericarditis, given its nonspecific clinical presentation and high mortality. Furthermore, it highlights the applicability of point-of-care ultrasonography in the diagnosis of the same.
